# Absolute and relative accelerometer thresholds for determining the association between physical activity and metabolic syndrome in the older adults: The Generation-100 study

**DOI:** 10.1186/s12877-017-0497-1

**Published:** 2017-05-16

**Authors:** Nina Zisko, Javaid Nauman, Silvana Bucher Sandbakk, Nils Petter Aspvik, Øyvind Salvesen, Trude Carlsen, Hallgeir Viken, Jan Erik Ingebrigtsen, Ulrik Wisløff, Dorthe Stensvold

**Affiliations:** 1 The K.G. Jebsen Center of Exercise in Medicine at Department of Circulation and Medical Imaging, Faculty of Medicine and Health Sciences, Norwegian University of Science and Technology, Trondheim, Norway; 2 Department of Sociology and Political Science, Faculty of Social Sciences and Technology Management, Norwegian University of Science and Technology, Trondheim, Norway; 3 Department of Cancer Research and Molecular Medicine, Faculty of Medicine and Health Sciences, Norwegian University of Science and Technology, Trondheim, Norway; 4 Department of Nursing Science, Faculty of Medicine and Health Sciences, Norwegian University of Science and Technology, Trondheim, Norway; 5 School of Human Movement & Nutrition Sciences, University of Queensland, Brisbane, Australia

**Keywords:** Actigraph, Oxygen uptake, Aging, VO_2peak_

## Abstract

**Background:**

When assessing population adherence to physical activity (PA) recommendation using accelerometers, absolute intensity threshold definition is applied despite having limited validity in those with low cardiorespiratory fitness (CRF), who are unable to reach them (e.g older adults). Thus, PA thresholds relative to CRF may be an alternative approach. We compared the proportion of the older adults meeting the PA recommendation when PA is assessed using absolute versus sex-and-CRF-adjusted (relative) accelerometer thresholds and determined the association between relative versus absolute moderate PA (MPA), vigorous PA (VPA) and moderate-to-vigorous PA (MVPA) and metabolic syndrome (MetS).

**Methods:**

Cross-sectional study of 509 men and 567 women aged 70–77. Accelerometer assessed MPA, VPA and MVPA were analyzed using absolute and relative thresholds. Meeting the PA-recommendation was defined as amounting ≥150 min/week in MPA/MVPA or 75 min/week in VPA, respectively. CRF was directly measured as peak oxygen uptake (VO_2peak_). MetS was defined as 3 or more of the following: elevated waist circumference, fasting glucose, hypertension, triglycerides, decreased HDL-cholesterol or diabetes, dyslipidemia or hypertension medication.

**Results:**

Higher proportion of the population met the recommendation when PA was assessed with relative compared to absolute thresholds: VPA (72.4% vs. 1.7%) and MVPA (75.2% vs. 33.8%). Logistic regression analysis revealed that men and women not meeting the relative-MVPA or VPA recommendation had higher likelihood of MetS (Men: MVPA OR: 1.59, 95% CI: 1.08–2.33. VPA OR: 1.81, 95%CI: 1.23–2.67 and Women: MVPA OR: 2.12, 95% CI: 1.36–3.31; VPA OR: 1.95, 95% CI: 1.29–2.95), compared to men and women meeting the relative MVPA or VPA recommendation. There was no significant association between MetS and absolute MVPA, MPA or VPA recommendations in the fully adjusted model.

**Conclusions:**

The association between meeting/not meeting the PA recommendation and MetS differed with method. Not meeting relative MVPA and VPA recommendation was associated with significantly higher likelihood for presence of MetS. Since relative intensity is part of the current PA recommendation, it should be considered when assessing population PA and associated health risks in the older adults.

**Trial registration:**

Clinical Trial Registration: NCT01931111 (Date of trial registration: July 19, 2013).

**Electronic supplementary material:**

The online version of this article (doi:10.1186/s12877-017-0497-1) contains supplementary material, which is available to authorized users.

## Background

More people die from cardiovascular disease (CVD) than from any other cause [1]. Since physical activity (PA) is important for cardiovascular health, all adults are recommended to perform ≥150 min of moderate or ≥75 min of vigorous PA weekly, or some combination of the two [2]. The PA intensity can be expressed as absolute or relative. Absolute intensity is quantified using work energy expenditure (i.e. metabolic equivalents-of-task or METs), while relative intensity is determined relative to individual cardiorespiratory fitness (CRF) (i.e. peak oxygen uptake or VO_2peak_) and differs for the unfit compared to fit individuals [3].

Accelerometers are often used to objectively assess population adherence to PA recommendation [4, 5]. Accelerometer output is given in counts [6]. However, the count thresholds used to define moderate-to-vigorous physical activity (MVPA) and assess PA recommendation adherence, are based on absolute intensity and are derived from physical exertion of healthy young to middle-aged adults [7]. These thresholds could have low validity in those with low CRF (i.e. older adults). As the CRF declines with age, it results in changes in relative effort required to perform PA [8, 9] and for many unfit older adults, absolute light intensity PA (<3 METs) requires moderate relative effort, while absolute vigorous intensity PA (6–9 METs) is often unattainable [8]. Those not meeting the absolute PA recommendation may be meeting the relative PA recommendation, likely resulting in underestimation of PA-recommendation adherence in this population.

It is estimated that 5.4 million people in the United Kindom would attain or exceed vigorous relative intensity (>70% of VO_2peak_) by walking at ≈4.8 km/h [10]. Therefore, even low absolute PA, if performed at high relative intensity, has potential to benefit many by improving CRF, which is a powerful predictor of mortality [11, 10]. However, relative PA assessment in a population is hindered by methodological limitations [12] and until recently, the only available relative thresholds were derived from physical exertion of young to middle-aged healthy adults. Further, methodology associated with application of these thresholds is rather complex, limiting their use to smaller studies [13].

Since PA recommendation is also given in relative intensity [2, 14], it may be valuable, in populations of varying CRF (i.e. older adults) to measure MVPA using recently published relative thresholds derived from physical exertion of the older adults [15]. Furthermore, it is not known if absolute or relative thresholds quantify MVPA that better associates with metabolic syndrome (MetS) [16], which was found to associate with CVD- and all-cause mortality in the older adults [17, 18] or what role the two components of MVPA, moderate (MPA) and vigorous (VPA) physical activity, play.

The aim of this study, therefore, was to compare the proportion of the older adults meeting relative versus absolute PA recommendation and to determine the method which quantifies PA that better associates with MetS.

## Methods

### Study participants

**Fig. 1:**
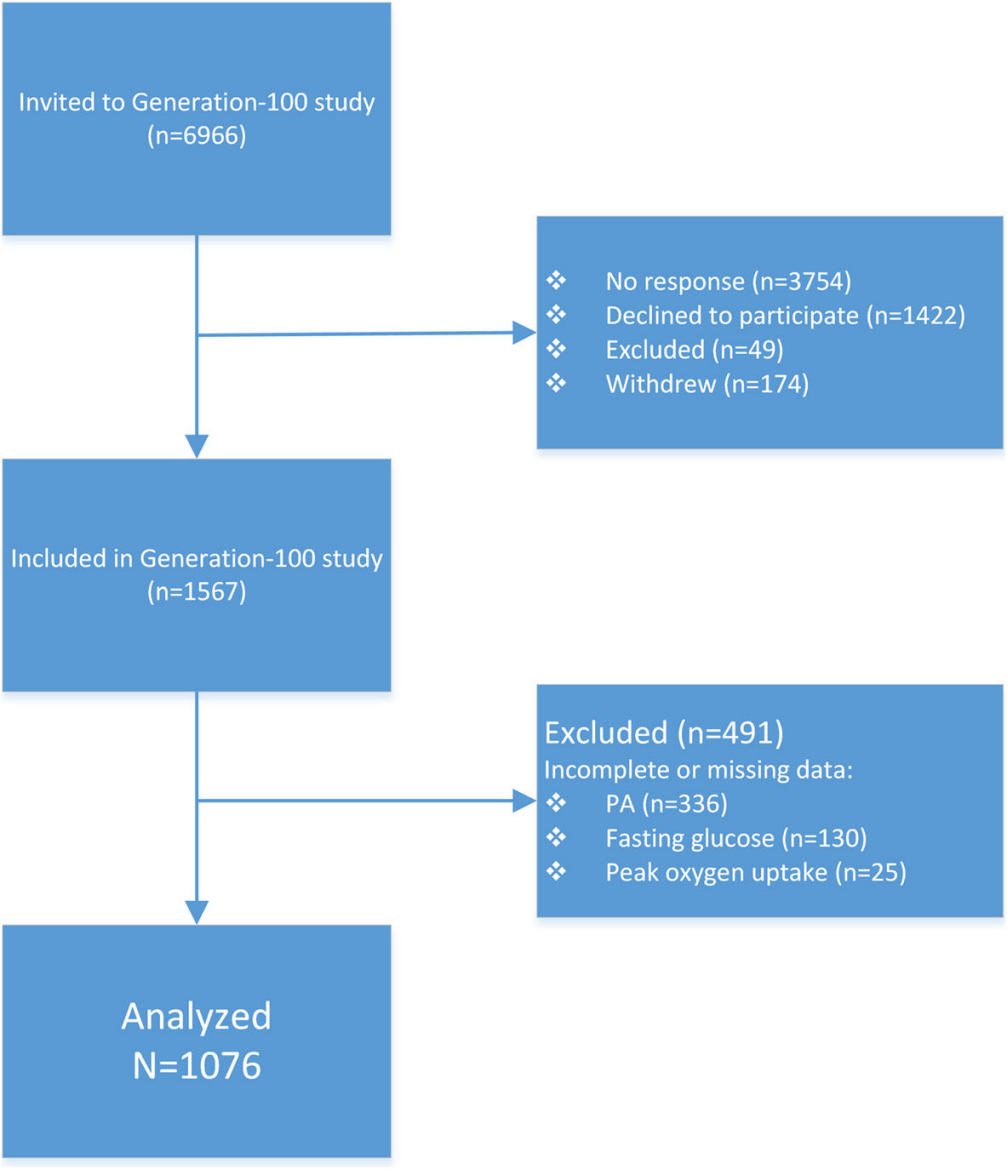
Study flowchart

This study is a part of the Generation 100 study, which aims to investigate the effect of exercise training on morbidity and mortality in the older adults. The Generation 100 study is described elsewhere [19] but briefly: 1567 of 6966 invited inhabitants of Trondheim (Norway), 70–77 years of age, fulfilled the inclusion criteria [19]. For the current study, we excluded participants with incomplete or missing PA (*n* = 336), fasting glucose (fasting time < 8 h) (*n* = 130), and VO_2peak_ (*n* = 25) data (Fig. 1). A total of 1076 (567 women) participants were included in the analyses.

All participants signed informed consent. The study was approved by the Regional Committee for Medical Research Ethics (2013/1609/REK Midt) and complied with the Declaration of Helsinki principles.

### Examinations

All examinations were conducted between August 2012 and June 2013. Detailed protocol is published elsewhere [19]. Briefly, participants were asked to come to the clinic on two separate days.

On day one, blood samples were taken and weight, height, waist-circumference and blood pressure were measured. Information on prescribed medication (hypertension, dyslipidemia and diabetes), alcohol, smoking status, and CVD (myocardial infarction, angina pectoris, heart failure, atrial fibrillation, other heart diseases and stroke) was obtained from a questionnaire [19].

On day two, VO_2peak_ was measured using ergospirometry employing an incremental protocol previously described elsewhere [19, 20]. Participants reporting CVD were tested using the American College of Sports Medicine/American Heart Association [21].

All participants were given Actigraph GT3X+ (Actigraph, Pensacola, USA), and were asked to wear it continuously for 7 consecutive days. Actigraph assesses acceleration, and hence PA, in three different axes. While vertical axis (VA) has been most utilized in research, tri-axial (VM) motion captures more complex movement [22, 23]. The VM model was found to better predict relative PA in the older adults than the VA-model and was for that reason used to quantify relative PA in this study [15]. The Actigraph output is given in counts per minute (CPM). The higher the CPM, the higher the estimated PA-intensity [24]. Each sample of data was summed over a 10-s *epoch*. Data between midnight and 6 am (6 h) and non wear time were excluded from the analysis. Non wear time was defined as intervals of zero counts lasting at least 60 consecutive minutes, with counts exceeding zero for no more than 2 min [5]. Participants with valid PA data of ≥10 h on ≥4 days were included in the analysis [5]. To quantify PA, the registered accelerometer time was categorized into intensity zones using previously published absolute [7] and relative thresholds [15] and time in different intensity zones was calculated by summing all minutes of PA above the respective thresholds. Briefly, the relative intensity MVPA (>62% of maximum heart rate), MPA (63–76% of maximum heart rate) and VPA (>77% of maximum heart rate) thresholds used in the current study were derived from and for the Generation 100 population [15]. To establish the relative thresholds, subjects from the Generation 100 study, wearing an Actigraph GT3+ model, walked/run on the treadmill while having submaximal and maximal oxygen uptake measured [15]. Relationship between maximum oxygen uptake %, maximum heart rate %, VM-CPM and sex was established using a mixed regression model. Detailed protocol of relative threshold derivation is published elsewhere [15]. The Freedson absolute intensity thresholds applied in the current study are an established method used to examine PA recommendation adherence [7]. Detailed protocol of Freedson absolute threshold derivation is published elsewhere [7]. All MVPA and MPA was analyzed in 10-min-bouts (with up to 2-min interruption allowance) and VPA in 5-min-bouts (with up to 1-min interruption allowance). All PA was wear-time adjusted by multiplying recorded PA time by 1080 min (24 h minus 6 h from midnight to 6 am) and dividing it by wear-time in minutes. PA analysis was done using Actilife 6.11.5 (Actigraph, Pensacola, USA).

### Data and statistical analysis

Descriptive data is presented as mean ± standard deviation for continuous and percentages for categorical variables. To test parameter differences between sexes of continuous variables, t-test was used. The chi square test was used to assess sex differences between categorical variables. Presence of at least three of the following five risk factors was defined as MetS: increased waist-circumference (≥80 cm in women and ≥94 cm in men); increased blood pressure (systolic ≥130 mmHg and/or diastolic ≥85 mmHg) or drug treatment for hypertension; decreased HDL-cholesterol (<1.3 mmol·L^−1^ in women and <1.0 mmol·L^−1^ in men) or drug treatment for dyslipidemia; increased triglycerides (≥1.7 mmol·L^−1^) or drug treatment for dyslipidemia; and increased fasting glucose (≥5.6 mmol·L^−1^) or drug treatment for diabetes [16]. Meeting the PA recommendations was classified based on the current American College of Sports Medicine/American Heart Association PA recommendations for older adults [2]. Henceforth, an accumulated time of ≥21.43 min/day (i.e. ≥150 min/week) spent at MPA and MVPA (in bouts of at least 10 min) and ≥10.71 min/day (i.e. ≥75 min/week) spent at VPA (in bouts of at least 5 min) was considered as meeting the current PA recommendations. The minutes spent in bouts of absolute and relative MVPA and MPA were summed up, and then dichotomized (below/meeting recommendation), so that those with ≥150 weekly minutes (21.4 min/day) were considered to have met the PA-recommendation. The minutes spent in bouts of VPA were summed up and dichotomized (below/meeting recommendation), so that those amounting ≥75 weekly minutes (or 10.7 min/day) were considered to have met the PA-recommendation. Logistic regression analyses were used to estimate the association between fulfillment of relative and absolute PA recommendation (i.e. below/meeting recommendation) and prevalence of MetS stratified by sex. Results are presented as odds ratios, and 95% confidence intervals (CI) were used to assess precision of estimates. Study participants that were meeting the PA recommendation were used as a reference in the logistic regression analysis. The basic PA model (model 1) was adjusted for age, with additional adjustment for smoking status, alcohol consumption, and history of CVD (model 2), with absolute PA additionally adjusted for CRF (model 3).

To test the robustness of our results, we performed a sensitivity analysis in those without (869 participants) and those with CVD (207 participants) in our cohort. To determine if the model improved the fit, a log-likelihood-ratio test was used. To compare the maximum likelihood models combining fit and complexity, we used the Bayesian-Information-Criterion (BIC) and Akaike-Information-Criterion (AIC) [25]. The smaller information criterion (AIC or BIC) denotes the model that fits the data better. All statistical tests were two-tailed and were performed using Stata (version 13.1 StataCorp.). The results were considered statistically significant if the *p*-value was less than 0.05.

## Results

### Characteristics of study participants, clinical measurements and questionnaires

The characteristics of study participant are shown in Table 1. The mean age was 72.5 ± 2.1 years. In total, 31.9% of our participants received drug treatment for hypertension, 6.0% for diabetes, and 8.9% for dyslipidemia. Eleven percent were obese (BMI > 30). Elevated waist circumference was the most predominant risk factor in both men and women with 77.9% and 77.4% prevalence, respectively. Second most predominant risk factor was elevated blood pressure and/or treatment for hypertension with 68.2% and 66.3% prevalence for men and women, respectively. Of the study sample, 38% were categorized as having MetS.

**Table 1:** Characterization of 509 men and 567 women aged 70–77 years participating in the study

Characteristics	All (*n* = 1076)	Men (*n* = 509)	Women (*n* = 567)
Age	72.5 ± 2.1	72.4 ± 2.1	72.5 ± 2.1
MetS^a^, (%)	38.0	42.8*	34.2
Waist circumference (cm)	89.3 ± 10	88.3 ± 10.7*	91.0 ± 10.8
Elevated waist circumference, (%)	80.5	77.9	77.4
Triglycerides (mmol**·**L^−1^)	1.13 ± 0.54	1.16 ± 0.57*	1.10 ± 0.52
Elevated triglycerides or drug treatment, (%)	20.6	24.8	16.9
HDL-cholesterol (mmol**·**L^−1^)	1.74 ± 0.50	1.56 ± 0.44*	1.90 ± 0.50
Reduced HDL-cholesterol, (%)	14.7	16.1	13.5
Blood pressure (mmHg)			
Systolic	133 ± 1	132 ± 16	134 ± 19
Diastolic	75 ± 9	77 ± 9*	73 ± 9
Elevated blood pressure or drug treatment (%)	67.2	68.2	66.3
Fasting glucose (mg**·**dL^−1^)	5.67 ± 0.88	5.85 ± 0.98*	5.51 ± 0.77
Elevated fasting glucose or drug treatment (%)	52.7	61.4	44.6
History of cardiovascular disease^b^ (%)	19.2	25.9*	13.2
Currently smoking (%)	7.6	8.3	7.0
Alcohol use (units∙week^−1^)	3.5 ± 3.7	4.2 ± 4.2*	2.9 ± 2.9
Absolute intensity moderate- to vigorous physical activity (min**·**day^−1^)	18.8 ± 20.1	19.5 ± 21.4	18.3 ± 18.8
Meeting absolute intensity moderate-to-vigorous physical activity recommendation (%)^c^	33.8	33.9	33.6
Relative intensity moderate- to vigorous physical activity (min**·**day^−1^)	44.0 ± 35.2	33.9 ± 27.3*	53.1 ± 38.9
Meeting relative moderate- to vigorous intensity physical activity recommendation (%)^c^	75.2	67.3*	82.2
Absolute intensity moderate physical activity (min**·**day^−1^)	16.8 ± 18.2	16.2 ± 18.3	17.3 ± 18.1
Meeting absolute moderate intensity physical activity recommendation (%)^c^	30.4	28.6	32.5
Relative intensity moderate physical activity (min**·**day^−1^)	2.6 ± 9.1	1.8 ± 6.3	3.3 ± 11.1
Meeting relative intensity moderate intensity physical activity recommendation (%)^c^	3.4	1.2*	5.6
Absolute intensity vigorous physical activity (min**·**day^−1^)	0.8 ± 3.7	1.4 ± 5.2*	0.1 ± 0.8
Meeting absolute vigorous intensity physical activity recommendation (%)^d^	1.7	3.4*	0.2
Relative intensity vigorous physical activity (min**·**day^−1^)	27.0 ± 23.2	24.1 ± 22.6*	29.8 ± 23.4
Meeting relative vigorous intensity physical activity recommendation (%)^d^	72.4	67.1*	77.4
Peak oxygen uptake (mL·kg^−1^·min^−1^)	28.9 ± 6.6	31.7 ± 6.7*	26.4 ± 5.1

Numbers are means ± standard deviation unless otherwise specified

*Statistically significant differences between sexes (*p* < 0.05)

^a^MetS: Metabolic syndrome risk factor clustering was defined as the presence of at least 3 of the following 5 risk factors: elevated waist circumference (being ≥80 cm in women and ≥94 cm in men); elevated triglycerides (being ≥1.7 mmol·L-1); reduced HDL-cholesterol (being ≤1.3 mmol·L-1 in women and ≤1.0 mmol·L-1 in men); elevated blood pressure (systolic ≥130 mmHg and/or diastolic ≥85 mmHg); and elevated fasting glucose (being ≥100 mg·dL-1).

^b^History of CVD was determined with current or former prevalence of myocardial infarction, angina pectoris, heart failure, atrial fibrillation, other heart diseases and/or stroke.

^c^Having ≥ 150 min per week moderate to vigorous or moderate intensity in bouts of at least 10 min = meeting absolute or relative moderate to vigorous or moderate PA recommendation

^d^Having ≥ 75 min per week in bouts of at least 5 min = meeting absolute or relative vigorous PA recommendation

No sex differences were observed for triglycerides, HDL-cholesterol, diastolic blood pressure, or in time spent in absolute MVPA. Men had higher waist-circumference, fasting glucose, history of CVD, alcohol consumption and VO_2peak_. Women spent more time in relative MVPA.

### Relative versus absolute intensity physical activity

Average accelerometer wear time was 16.1 ± 1.1 h per day. Four, five and six or more valid days of PA data were available for 1.4%, 7.2% and 91.4% of the participants, respectively. Average time in bouts of absolute MVPA for men and women was 19.5 ± 21.4, and 18.3 ± 18.8 min per day, respectively, while time spent in relative MVPA bouts was 33.9 ± 27.3 and 53.1 ± 38.9 min per day for men and women, respectively. The proportion of men and women meeting the absolute MVPA recommendation did not differ, while a difference between sexes was observed with relative MVPA recommendation. Significantly higher proportion (40%) of the population met the relative versus absolute MVPA recommendation. Majority of women (82.2%) amounted ≥150 weekly minutes in relative MVPA, with only 33.6% doing so in absolute MVPA. Higher proportion of men (33%) met the relative MVPA compared to absolute MVPA recommendation. Men and women spent significantly more time in relative compared to absolute VPA and significantly higher proportion of the population met the relative compared to absolute VPA recommendation, while significantly more time was spent in absolute compared to relative MPA and higher proportion of the population met absolute versus relative MPA recommendation (Table 1).

### Metabolic syndrome

**Fig. 2:**
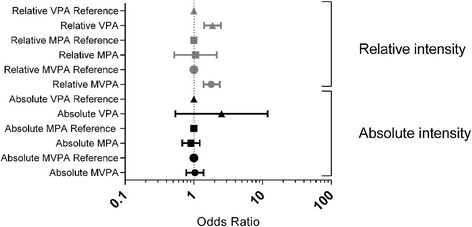
Adjusted odds ratio for the prevalence of MetS according to adherence to absolute versus relative intensity PA-recommendation among 1076 men and women aged 70–77 years. Study participants that were meeting the PA-recommendation were used as a reference in the logistic regression analysis. The model for relative and absolute PA was adjusted for age, smoking status, alcohol consumption and history of CVD with absolute PA model additionally adjusted for CRF

Not meeting the relative MVPA recommendation was associated with more than twofold (OR: 2.12, 95% CI: 1.36–3.31) and 59% (OR: 1.59, 95% CI: 1.08–2.33) higher likelihood for having MetS in women and men, respectively, when compared to meeting the relative MVPA-recommendation (Table 2). Similarly, not meeting the relative VPA recommendation was associated with 81% (OR: 1.81, 95% CI: 1.23–2.67) and 95% (OR: 1.95, 95%CI: 1.29–2.95) higher likelihood for having MetS in men and women, respectively. There was no significant association between MetS and absolute MVPA, MPA or VPA recommendations in the fully adjusted model (Table 3, Fig. 2).

**Table 2:** Adjusted odds ratios (OR; 95% confidence interval) for the prevalence of MetS according to meeting or not meeting physical activity measured objectively using relative intensity accelerometer thresholds among 509 men and 567 men aged 70–77 years

	Men				Women			
	MetS^c^				MetS^c^			
	Yes	No	OR (95% CI)^a^	OR (95% CI)^b^	Yes	No	OR (95% CI)^a^	OR (95% CI)^b^
Relative MVPA^d^ recommendation								
Below	86	80	1.74 (1.19–2.53)	1.59 (1.08–2.33)	49	52	2.15 (1.39–3.33)	2.12 (1.36–3.31)
Meeting	132	211	1.00 (Reference)	1.00 (Reference)	142	324	1.00 (Reference)	1.00 (Reference)
Relative MPA recommendation								
Below	215	287	1.00 (0.22–4.54)	0.87 (0.18–4.09)	182	355	1.19 (0.53–2.66)	1.11 (0.49–2.51)
Meeting	3	4	1.00 (Reference)	1.00 (Reference)	9	21	1.00 (Reference)	1.00 (Reference)
Relative VPA^e^ recommendation								
Below	91	76	2.02 (1.39–2.95)	1.81 (1.23–2.67)	57	67	1.96 (1.30–2.95)	1.95 (1.29–2.95)
Meeting	127	215	1.00 (Reference)	1.00 (Reference)	134	309	1.00 (Reference)	1.00 (Reference)

*CI* confidence interval.

^a^Model 1: Adjusted for age.

^b^Model 2: Adjusted for age, smoking status, alcohol consumption, history of cardiovascular disease.

^c^MetS: Metabolic syndrome risk factor clustering was defined as the presence of at least 3 of the following 5 risk factors: elevated waist circumference (being ≥80 cm in women and ≥94 cm in men); elevated triglycerides (being ≥1.7 mmol·L-1); reduced HDL-cholesterol (being ≤1.3 mmol·L-1 in women and ≤1.0 mmol·L-1 in men); elevated blood pressure (systolic ≥130 mmHg and/or diastolic ≥85 mmHg); and elevated fasting glucose (being ≥100 mg·dL-1).

^d^bouts of at least 10 min.

^e^bouts of at least 5 min.

**Table 3:** Adjusted odds ratios (OR; 95% confidence interval) for the prevalence of MetS according to meeting or not meeting physical activity measured objectively using absolute intensity accelerometer thresholds among 509 men and 567 men aged 70–77 years

	Men					Women				
	MetS^d^					MetS^d^				
	Yes	No	OR (95% CI)^a^	OR (95% CI)^b^	OR (95% CI)^c^	Yes	No	OR (95% CI)^a^	OR (95% CI)^b^	OR (95% CI)^c^
Absolute MVPA^e^ recommendation										
Below	158	178	1.68 (1.15–2.46)	1.69 (1.15–2.50)	1.36 (0.90–2.06)	135	241	1.35 (0.93–1.97)	1.32 (0.90–1.94)	0.77 (0.5–1.17)
Meeting	60	113	1.00 (Reference)	1.00 (Reference)	1.00 (Reference)	56	135	1.00 (Reference)	1.00 (Reference)	1.00 (Reference)
Absolute MPA recommendation										
Below	160	206	1.14 (0.77–1.69)	1.20 (0.80–1.79)	1.09 (0.71–1.65)	135	245	1.29 (0.88–1.88)	1.26 (0.86–1.85)	0.75 (0.49–1.15)
Meeting	58	85	1.00 (Reference)	1.00 (Reference)	1.00 (Reference)	56	131	1.00 (Reference)	1.00 (Reference)	1.00 (Reference)
Absolute VPA^f^ recommendation										
Below	216	275	6.43 (1.46–28.35)	6.16 (1.37–27.57)	2.88 (0.61–13.68)	191	376	-	-	-
Meeting	2	16	1.00 (Reference)	1.00 (Reference)	1.00 (Reference)	0	0	-	-	-

*CI* confidence interval.

^a^Model 1: Adjusted for age.

^b^Model 2: Adjusted for age, smoking status, alcohol consumption, history of cardiovascular disease.

^c^Model 3: Adjusted for age, smoking status, alcohol consumption, history of cardiovascular disease and cardiorespiratory fitness (measured as VO2peak).

^d^MetS: Metabolic syndrome was defined as the presence of at least 3 of the following 5 risk factors: elevated waist circumference (being ≥80 cm in women and ≥94 cm in men); elevated triglycerides (being ≥1.7 mmol·L-1); reduced HDL-cholesterol (being ≤1.3 mmol·L-1 in women and ≤1.0 mmol·L-1 in men); elevated blood pressure (systolic ≥130 mmHg and/or diastolic ≥85 mmHg); and elevated fasting glucose (being ≥100 mg·dL-1).

^e^bouts of at least 10 min.

^f^bouts of at least 5 min.

The log-likelihood-test was significant (*p* < 0.001), and the fully adjusted model for relative PA was a better fit on the basis of lower AIC and BIC values (absolute MVPA: AIC: 1424.3, BIC: 1434.2 versus relative MVPA: AIC: 1409.89, BIC: 1419.852). The sensitivity analysis of those without CVD produced similar results, indicating 69% (OR: 1.69, 95% CI: 1.21–2.36, Additional file 1: Table S1) higher likelihood for MetS in those not meeting the relative MVPA recommendation and 88% (OR: 1.88, 95% CI: 1.36–2.60, Additional file 1: Table S1) higher likelihood of MetS in those not meeting the relative VPA recommendation. In those reporting CVD, not meeting the relative MVPA recommendation was associated with more than twofold higher likelihood (OR: 2.30, 95%CI: 1.23–4.31, Table 1) of MetS and 85% (OR: 1.85 95%CI: 1.02–3.36, Additional file 1: Table S1) higher likelihood of MetS in those not meeting the relative VPA recommendation. No significant association was observed in those with or without CVD not meeting the absolute MVPA recommendation and MetS (Additional file 1: Table S2).

## Discussion

The main finding of the current study was that a significantly higher proportion of our older adults met the relative versus absolute PA recommendation. Those below the relative PA recommendation were more likely to have increased prevalence of MetS compared to those below the absolute PA recommendation. Importantly, not meeting the relative VPA recommendation was associated with higher likelihood of MetS, while no such association was observed in those below the absolute MPA, VPA or MVPA recommendation.

To our knowledge, this is the first study utilizing accelerometers to objectively assess relative PA in a large population of older adults using thresholds stratified for sex and CRF, and investigating how the relative MVPA and its two components, MPA and VPA, associate with MetS. As individuals age, their CRF declines, producing a change in the relative intensity of effort required for PA [8, 9]. Many individuals with low CRF, including the older adults, require moderate relative effort for low absolute PA and can rarely reach absolute MPA, MVPA or VPA [8].

Previous studies showed accelerometer thresholds to vary with age, with older individuals having lower accelerometer count output. This difference was ascribed to variation in CRF, which stressed the importance of relative intensity when assessing PA in populations of different ages and CRF levels [26]. Ozemek et al. were the first to illustrate the importance of CRF in accelerometer PA assessment when they showed an unfit younger individual to have a significantly lower MVPA threshold compared to a more fit older individual [13]. Notably, they found the MVPA counts to be significantly correlated to CRF, explaining ca. 30% of the variability, while only ca. 1% could be explained by age and BMI. However, Ozemek’s approach to relative PA assessment is based on physical exertion of younger adults and requires complex methodology, making its use cumbersome in larger studies [13]. In our study we utilized thresholds derived from physical exertion of older adults to quantify relative PA [15]. These thresholds are adjusted for CRF and sex and are feasible to use in large studies [15].

Higher proportion of our participants met the absolute MVPA recommendation compared to other studies on older Norwegians [27–29]. This difference could be ascribed to fewer study participants [27–29], wider age interval [27–29], and different methodology [27–29] of these studies. For instance, these studies utilized the Troiano [27–29], while we used the Freedson threshold [27–29] for absolute MVPA assessment and they applied an earlier (30 min of daily MVPA) recommendation, while we used the current Norwegian PA recommendation [27–29]. Notably, by applying the earlier PA recommendation in a preliminary analysis of our study, we obtained a similar result, with 23% of our population meeting the absolute MVPA recommendation.

The lack of association between absolute PA recommendation adherence and MetS in the current study may be attributable to absolute thresholds being too high for this population. Some older adults may have difficulties reaching absolute thresholds and as a result fail to meet the absolute PA recommendation. In fact, results of our absolute VPA analysis show that out of 509 men and 567 women included in our study, only two individuals, both men, managed to meet the absolute VPA recommendation. One could argue that expecting unfit individuals, such as some older adults, to accumulate sufficient time at an absolute intensity higher than their maximal capacity (e.g. VO_2peak_ ~ 3METs) in order to meet the PA recommendation is unrealistic. Relative thresholds, on the other hand, are individualized in terms of CRF and sex. Indeed, it has been shown that the least fit individuals from the Generation 100 cohort are also least likely to adhere to both absolute and relative PA recommendation compared to the moderately fit and highly fit individuals [30]. Approximately 30% of our population fail to meet the relative PA recommendation. This is important information as it may allow researchers to identify the least active individuals when designing strategies to increase PA participation in older adults. Our results encourage researchers to consider PA intensity in both relative and absolute terms when utilizing accelerometers in assessment of PA, especially in populations with varying degrees of fitness and physical functionality. Thus, meeting the relative PA recommendation for older adults may not only prove a realistically achievable goal, but may be beneficial for health.

The strength of our study is that it uses objective measures of absolute and relative PA, and relates them to objectively measured health indicator in a large sample of older adults. Furthermore, CRF of our population was assessed objectively. However, relative MPA did not perform well in our study. This was likely due to 2-min interruption allowance during the 10-min-bout data processing. Actilife defines interruptions as “minutes outside of the minimum and maximum count levels” [31]. These interruptions do not distinguish peaks from drops. Unlike the MVPA and VPA (with only drops counted as interruptions), the MPA threshold is defined as a range where peaks and drops crossing the range are added up, resulting in higher number of discounted bouts.

The extensive analysis of non-participants in our study revealed presence of a selection bias, with the participants reporting higher PA, education and better health than non-participants [19]. Our participants were likely fitter than non-participants. Nevertheless, our population was diverse and included healthy as well as older adults with co-morbidities [19]. Our population is a good representation of the general older Norwegian population, with similar co-morbidity prevalence as described in the 2015 Norwegian Institute of Public Health report [19, 32]. The thresholds for relative PA analysis were sex and CRF stratified, and created specifically for our population. However, the external generalizability of our findings to populations of different ages and ethnicities is limited, and the cross-sectional nature of our study prevents us from establishing causality. Therefore, longitudinal studies on more diverse populations with hard endpoints such as morbidity and mortality are needed.

## Conclusion

In conclusion, higher proportion of our sample of older adults met relative versus absolute PA recommendation. Not meeting the relative PA recommendation associated with higher likelihood for having MetS, with no such association observed when not meeting the absolute PA recommendation. Since relative intensity is part of the current PA recommendation, it should be considered when assessing population PA and associated health risks.

## Additional file

Additional file 1: Table S1. Adjusted odds ratios (OR; 95% confidence interval) for the prevalence of MetS according to meeting or not meeting physical activity recommendation measured objectively using relative intensity accelerometer thresholds among 869 participants 70–77 year-old without cardiovascular disease (CVD). **Table S2.** Adjusted odds ratios (OR; 95% confidence interval) for the prevalence of MetS according to meeting or not meeting physical activity recommendation measured objectively using absolute intensity accelerometer thresholds among 869 participants 70–77 year-old without cardiovascular disease (CVD). (DOCX 16 kb)

## Abbreviations

CRF: Cardiorespiratory fitness
MetS: Metabolic syndrome
MPA: Moderate physical activity
MVPA: Moderate to vigorous physical activity
PA: Physical activity
VO2peak: Peak oxygen uptake
VPA: Vigorous physical activity
