# Supplementary material for: Absolute and relative accelerometer thresholds for determining the association between physical activity and metabolic syndrome in the older adults: The Generation-100 study
Source: BMC Geriatr. 2017 May 16;17:109. doi: 10.1186/s12877-017-0497-1 (PMC5433020; doi:10.1186/s12877-017-0497-1)
Supplement: Additional file 1: Table S1. — Adjusted odds ratios (OR; 95% confidence interval) for the prevalence of MetS according to meeting or not meeting physical activity recommendation measured objectively using relative intensity accelerometer thresholds among 869 participants 70–77 year-old without cardiovascular disease (CVD). Table S2. Adjusted odds ratios (OR; 95% confidence interval) for the prevalence of MetS according to meeting or not meeting physical activity recommendation measured objectively using absolute intensity accelerometer thresholds among 869 participants 70–77 year-old without cardiovascular disease (CVD). (DOCX 16 kb) [file 12877_2017_497_MOESM1_ESM.docx]

**Table S1.** Adjusted odds ratios (OR; 95% confidence interval) for the prevalence of MetS according to meeting or not meeting physical activity recommendation measured objectively using relative intensity accelerometer thresholds among 869 participants 70-77 year-old without cardiovascular disease (CVD).

|  |  | **No CVD** | | | |  | **With CVD** | | | |
| --- | --- | --- | --- | --- | --- | --- | --- | --- | --- | --- |
|  |  | **MetS ^§^** | |  | |  | **MetS ^§^** | |  | |
|  |  | **Yes** | **No** | **OR (95% CI)^*^** | **OR (95% CI)^†^** |  | **Yes** | **No** | **OR (95% CI)^*^** | **OR (95% CI)^†^** |
| **Relative MVPA^a^ recommendation** |  |  | | | |  |  |  |  |  |
| Below |  | 86 | 105 | 1.72 (1.23-2.40) | 1.69 (1.21-2.36) |  | 49 | 27 | 2.19 (1.20-4.00) | 2.30 (1.23-4.31) |
| Meeting |  | 216 | 462 | 1.00 (Reference) | 1.00 (Reference) |  | 58 | 73 | 1.00 (Reference) | 1.00 (Reference) |
| **Relative MPA recommendation** |  |  | | | |  |  |  |  |  |
| Below |  | 292 | 542 | 1.28 (0.60-2.72) | 1.26 (0.59-2.69) |  | 105 | 100 | - | - |
| Meeting |  | 10 | 25 | 1.00 (Reference) | 1.00 (Reference) |  | 2 | 0 | 1.00 (Reference) | 1.00 (Reference) |
| **Relative VPA^b^ recommendation** |  |  | | | |  |  |  |  |  |
| Below |  | 96 | 110 | 1.90 (1.38-2.62) | 1.88 (1.36-2.60) |  | 67 | 33 | 1.82 (1.02-3.24) | 1.85 (1.02-3.36) |
| Meeting |  | 206 | 457 | 1.00 (Reference) | 1.00 (Reference) |  | 52 | 55 | 1.00 (Reference) | 1.00 (Reference) |

^*^ Model 1: Adjusted for age.

^†^ Model 2: Adjusted for age, smoking status, alcohol consumption.

^§^ MetS:Metabolic syndrome was defined as the presence of at least 3 of the following 5 risk factors: elevated waist circumference (being ≥80 cm in women and ≥94 cm in men); elevated triglycerides (being ≥1.7 mmol**·**L^-1^); reduced HDL-cholesterol (being ≤1.3 mmol**·**L^-1^ in women and ≤1.0 mmol**·**L^-1^ in men); elevated blood pressure (systolic ≥130 mmHg and/or diastolic ≥85 mmHg); and elevated fasting glucose (being ≥100 mg·dL^-1^).

CI, confidence interval.

^a^bouts of at least 10 min

**Table S2.** Adjusted odds ratios (OR; 95% confidence interval) for the prevalence of MetS according to meeting or not meeting physical activity recommendation measured objectively using absolute intensity accelerometer thresholds among 869 participants 70-77 year-old without cardiovascular disease (CVD).

|  |  | **No CVD** | | | | |  | **With CVD** | | | | |
| --- | --- | --- | --- | --- | --- | --- | --- | --- | --- | --- | --- | --- |
|  |  | **MetS ^§^** | |  | | |  | **MetS ^§^** | |  | | |
|  |  | **Yes** | **No** | **OR (95% CI)^*^** | **OR (95% CI)^†^** | **OR (95% CI)** ^‡^ |  | **Yes** | **No** | **OR (95% CI)^*^** | **OR (95% CI)^†^** | **OR (95% CI)** ^‡^ |
| **Absolute MVPA^a^ recommendation** |  |  | | | |  |  |  |  |  |  |  |
| Below |  | 217 | 346 | 1.64 (1.21-2.22) | 1.62 (1.20-2.20) | 1.13 (0.81-1.57) |  | 76 | 73 | 1.64 (1.21-2.22) | 1.62 (1.20-2.20) | 1.13 (0.81-1.57) |
| Meeting |  | 85 | 221 | 1.00 (Reference) | 1.00 (Reference) | 1.00 (Reference) |  | 31 | 27 | 1.00 (Reference) | 1.00 (Reference) | 1.00 (Reference) |
| **Absolute MPA recommendation** |  |  | | | |  |  |  |  |  |  |  |
| Below |  | 219 | 377 | 1.31 (0.96-1.78) | 1.30 (0.95-1.77) | 0.99 (0.71-1.37) |  | 76 | 74 | 0.94 (0.50-1.76) | 0.95 (0.50-1.82) | 0.73 (0.37-1.44) |
| Meeting |  | 83 | 190 | 1.00 (Reference) | 1.00 (Reference) | 1.00 (Reference) |  | 31 | 26 | 1.00 (Reference) | 1.00 (Reference) | 1.00 (Reference) |
| **Absolute VPA^b^ recommendation** |  |  | | | |  |  |  |  |  |  |  |
| Below |  | 302 | 551 | - | - | - |  | 105 | 100 | - | - | - |
| Meeting |  | 0 | 16 | 1.00 (Reference) | 1.00 (Reference) | 1.00 (Reference) |  | 2 | 0 | 1.00 (Reference) | 1.00 (Reference) | 1.00 (Reference) |

^*^Model 1: Adjusted for age.

^†^ Model 2: Adjusted for age, smoking status, alcohol consumption.

^‡^ Model 3: Adjusted for age, smoking status, alcohol consumption and cardiorespiratory fitness (measured as VO_2_peak).

^§^ MetS: Metabolic syndrome was defined as the presence of at least 3 of the following 5 risk factors: elevated waist circumference (being ≥80 cm in women and ≥94 cm in men); elevated triglycerides (being ≥1.7 mmol**·**L^-1^); reduced HDL-cholesterol (being ≤1.3 mmol**·**L^-1^ in women and ≤1.0 mmol**·**L^-1^ in men); elevated blood pressure (systolic ≥130 mmHg and/or diastolic ≥85 mmHg); and elevated fasting glucose (being ≥100 mg·dL^-1^).

CI, confidence interval.

^a^bouts of at least 10 min.
